# Tin Foil Fillings

**Published:** 1894-01

**Authors:** 


ARTICLE IV.

### TIN FOIL FILLINGS.

What dentist of the present day employs tin foil regularly in his practice? Echo alone answers. And yet it is one of the best materials at our command. It possesses inherent qualities which make it the ideal filling for a certain class of cavities. Some of the older dentists can remember the time when it was extensively employed by men whose operations stood the crucial test of many years hard service. Why then has it fallen into disuse? Is it because its proper insertion requires a skill of too high order? Is it because the dentists of the present day have grown indolent, and seek their own ease more than they do the highest good of their patients? Either of these reasons would not be very creditable to us as a body, and yet, no other that seems sufficient readily presents itself to the mind.

The use of tin foil is not usually taught in our schools, or at least it is not given the importance which of right belongs to it. It is so easy to putty up a cavity with a plastic filling, that amalgam has been made to take its place, and yet, when properly inserted in a cavity to which it is adapted, it is infinitely superior. All plastic fillings which depend for their hardness upon crystallization subsequent to their insertion, are liable to the changes in form which that process necessarily entails. Expansion or contraction unavoidably follow. When water crystallizes into ice there is a decided expansion. The general rule for metals is that they contract. Aside from this, there is usually a change of form during crystallization, so that a crystallizing body will not be of the exact shape of the matrix in which it is placed.

If an amalgam be inserted in a cylinderical hole in a piece of ivory, and its surface carefully leveled, it will usually be found that when it has become entirely set the edges will have drawn away from the matrix, and the surface will be raised in the center, so that it will bear considerable filing before it is again level with the matrix, and then a minute channel will be found all about its periphery. This change is something inherent to the process of crystallization.

From this defect tin foil is free. When once well inserted it stays in precisely the same condition. It is soft and ductile and can be easily and perfectly adapted to the walls of a cavity. At the same time, when thoroughly condensed, it has nearly the wearing qualities of gold. A bar of pure tin has almost the impenetrability of the nobler metal. It can be worked very quickly, and a minute quantity of moisture is not as fatal as to a gold filling. It is peculiarly congenial to tooth tissue, and seems at times to exercise a decided therapeutic influence upon it. There would seem, then, to be no reason why dentists who are seeking for the best results should not employ it largely, unless it be that they are ignorant of its worth, or of the best methods of working it.

The average gold worker, when he attempts to use tin foil, seeks to employ the same kind of manipulation with it that he does with gold foil. But the character of the two metals are widely at variance. Gold can be made to cohere. One particle can be added to another, and actually welded to it. This is not the case with tin. It does not weld, and the most that can be done in consolidating it is to so intermingle the surface of two pieces that they seem practically to be one. A pellet of thin gold foil can be laid upon a bar of the same metal, and by the impact of a smooth instrument united to it. Not so with tin. Under like circumstances it would be found necessary to use a sharp-pointed instrument, and by a succession of indentations to drive the particles of the one piece into the other. Hence the impact of a mallet is out of place in consolidating tin. It is impossible to add pellet to pellet, and by hammering with a mallet to build up a filling, as with gold.

The best way to insert tin is to use it in the form of cylinders, following the instructions laid down by the early operators for using soft gold. In the early days dentists were unacquainted with the welding properties of gold foil, and depended upon wedging it in. That is precisely the manipulation proper for tin foil, which has the qualities that gold was formerly thought to possess. Cylinders of different sizes should be preapared by rolling strips of folded tin foil about a smooth wire. These are then placed on end in the cavity to be filled, and pressed laterally, toward the periphery, the end being allowed to project. Another cylinder is added and pressed toward the margins, and this process is continued until the walls are completely lined by the cylinders. More are added in the center until the cavity is filled. Then with a sharp-pointed excavator, or a plugger of analagous shape, the surface is examined to see if there be any imperfections. If there are, the instrument is worked down into the filling, lateral pressure being almost exclusively used. When the hole is sufficient another small cylinder is inserted, and this is continued until the instrument can no longer be worked into the filling.

Then commences the consolidation of it by means of serrated condensers. For the first time pressure nearly in the direction of the axis of the tooth is employed. Heretofore all pressure has been lateral. By hand-pressure the surface is gradually condensed down, the projecting cylinders forming the surplus material until a dense filling is the result. It must be understood that nothing save the ends of the cylinders are presented. In no case should the tin be arranged in layers, for they are likely to flake off. If there is a deficiency of material at any point, more tin should be added by working a hole into which another cylinder may be inserted with a projecting end, which subsequently can be condensed down. The mallet should not be used, because under its blows the tin is literally chopped out. If a filling inserted in this manner be ground down upon its surface with a corundum stone, its density and impenetrability will surprise many experienced dentists.

Of course the class of cavities to which tin is best adapted are those with four walls, but a compound cavity, involving the crown and approximate surface, may be readily filled with tin, by so arranging the cylinders that their ends shall be presented wherever the filling is exposed. But two kinds of pluggers are needed, wedge-shaped ones for inserting and pressing to place the cylinders, and round or square condensers for consolidating the surface.—*The Dental Practitioner and Advertiser.*

